# Host Mucosal Niche and Rearing Environment Are Associated with Distinct Gut and Gill Microbiota of *L. crocea* (*Larimichthys crocea*)

**DOI:** 10.3390/vetsci13070710

**Published:** 2026-07-19

**Authors:** Ding Li, Xiaoping Wu, Fengfang Zhou, Jiacheng Zhang, Kuncan Wei, Yulin Lu, Fangyu Yuan, Weiqing Huang

**Affiliations:** 1 College of Marine Sciences, Ningde Normal University, Ningde 352100, Chinazhangjiacheng68@163.com (J.Z.); weikc0421@163.com (K.W.); luyulin0925@163.com (Y.L.); 2 Fujian Provincial University Engineering Research Center for Deep Processing of Mindong Aquatic Products, Ningde 352100, China; 3 College of Biological Science and Engineering, Ningde Normal University, Ningde 352100, China; zhoufengfang123@163.com; 4 School of Medicine, Fuzhou University, Fuzhou 350108, China

**Keywords:** *Larimichthys crocea*, gut microbiota, gill microbiota, saline–alkaline aquaculture

## Abstract

Microbial communities associated with fish mucosal tissues play important roles in host health and environmental adaptation. However, the effects of contrasting aquaculture conditions on the microorganisms of the large yellow croaker (*Larimichthys crocea*, *L. crocea*) remain poorly understood. In this study, 16S rRNA gene sequencing was used to compare the gut and gill microbiota of *L. crocea* cultured in a marine system and a saline–alkaline system, together with the corresponding surrounding water samples. The results showed that both host tissue type and rearing environment significantly influenced microbial community structure. Water samples generally exhibited higher alpha diversity than host-associated samples, whereas the gut and gill communities were clearly separated from the surrounding water microbial community. The intestine showed stronger culture system differentiation than the gill, suggesting greater niche selectivity, while the gill retained a closer association with the rearing water. Across multiple taxonomic levels, microbial composition differed markedly among water, gut, and gill samples, and the divergence between the marine culture and saline–alkaline culture system became more pronounced at finer taxonomic resolution. Differentially enriched taxa further revealed clear tissue- and habitat-associated microbial patterns. In addition, several dominant taxa were significantly correlated with environmental variables, especially salinity and nutrient-related factors, indicating that physicochemical heterogeneity contributed substantially to microbial community assembly. Overall, this study shows that host mucosal niche and rearing environment are associated with distinct gut and gill microbiota patterns in *L. crocea* and provides preliminary ecological information for future microbiome-informed aquaculture management, functional validation of candidate taxa, and the development of land-based culture systems for marine fish.

## Simple Summary

The gut and gill are important mucosal tissues that help fish interact with their internal and external environments. However, it remains unclear how these two microbial habitats respond to different aquaculture conditions. In this study, we compared the gut, gill, and rearing-water microbiota of large yellow croaker cultured in a coastal marine system in Ningde and an indoor saline–alkaline water system in Ningxia. The results showed that the gut and gill contained distinct microbial communities and differed from the surrounding water. The gut microbiota showed stronger differentiation between the two rearing systems, suggesting greater host-related selection, whereas the gill microbiota retained a stronger association with environmental conditions. Salinity, alkalinity, and nutrient-related variables were associated with microbial community variation. These findings improve our understanding of how host mucosal tissues and rearing environments jointly shape the microbiota of large yellow croaker and provide useful information for microbiome-informed management in different aquaculture systems.

## 1. Introduction

Fish-associated microbiota are integral components of the aquatic holobiont and contribute to nutrient metabolism, mucosal homeostasis, immune regulation, and resistance to pathogens [1]. In recent years, the fish microbiome has become an important research focus in aquaculture because microbial communities are closely linked to host health, growth performance, and environmental adaptation [2]. Previous studies have shown that fish-associated microbial assemblages are not simple reflections of the surrounding environment but are shaped by the combined effects of host biology, habitat, diet, and water physicochemical conditions [3].

Among host-associated microbial habitats, the intestine and gill are two particularly important but ecologically contrasting mucosal niches. In the present study, the term “host mucosal niche” refers specifically to tissue-level microbial habitats within the fish host, namely, the intestine and the gill. These two tissues were examined simultaneously because they represent different degrees of host control and environmental exposure within the same fish species. The gut microbiota is closely associated with digestion, nutrient utilization, and immune function, whereas the gill microbiota forms a frontline interface between the fish and the surrounding aquatic environment [4]. Because the gill is continuously exposed to external water, it is often more environmentally responsive than the intestine, while the gut is usually subject to stronger host filtering. This paired comparison therefore provides a useful framework for distinguishing microbial patterns primarily associated with internal host filtering from those more strongly linked to direct environmental exposure. This tissue-specific differentiation has been reported in several fish species, indicating that different body sites can harbor distinct microbial assemblages with different ecological sensitivities [5].

In addition to host tissue specificity, environmental heterogeneity is a major driver of microbial community assembly in fish. Factors such as salinity, temperature, nutrient availability, and rearing system have all been shown to influence fish-associated microbiota [6]. In particular, salinity is widely recognized as an important ecological filter in aquatic systems and has been reported to significantly affect gut microbial composition in cultured fish [7]. Comparative studies in aquaculture ecosystems have further shown that host-associated microorganisms are simultaneously influenced by surrounding water communities and host-mediated selection, resulting in dynamic but non-random microbial structures under different farming conditions [8].

*L. crocea* is one of the most economically important marine fish species in China and has long been a representative species in Chinese mariculture [9]. At the same time, it is also a key target species in the development of modern aquaculture systems [10]. Previous studies on *L. crocea* have shown that its gut microbiota differs from feed and culture water, and recent work has further demonstrated that the skin, gill, and gut of this species harbor distinct microbial communities that respond differently to environmental variation [11]. However, despite these advances, current studies have mainly focused on temporal variation, age effects, or conventional marine farming systems, whereas the microbial responses of *L. crocea* under contrasting rearing environments remain poorly understood [12]. These contrasts include differences in temperature; salinity; alkalinity; pH; dissolved oxygen; nutrient conditions such as NO_2_^−^, NO_3_^−^, NH_4_^+^, and PO_4_^3−^; and total phosphorus.

In recent years, land-based culture of marine fish has attracted increasing attention because it may provide new opportunities for the expansion of aquaculture space, improvement of environmental control, and support of sustainable fisheries development. Under such conditions, saline–alkaline water culture systems represent a distinct ecological background compared with conventional coastal seawater farming, and may impose different selective pressures on both environmental microorganisms and host-associated microbial communities [6]. Understanding how these different rearing environments influence the microorganism of *L. crocea* is therefore important not only for microbial ecological research but also for probiotic screening, aquaculture health management, and the optimization of the “marine fish cultured on land” model [5].

In this study, we did not aim merely to determine whether microbial communities differed among tissues or between regions, as such differences have been widely reported in fish microbiome studies. Instead, we focused on how host mucosal niche selectivity and environmental exposure jointly structure the gut and gill microbiota of *L. crocea* under two contrasting aquaculture contexts: a conventional marine culture system in Ningde and a saline–alkaline land-based culture system in Ningxia. We hypothesized that the intestine, as a more internally regulated mucosal habitat, would exhibit stronger host filtering and greater marine culture/saline–alkaline culture system differentiation, whereas the gill, as a respiratory and osmoregulatory interface directly exposed to the surrounding water, would retain a stronger environmental signal. By analyzing gut, gill, and corresponding water microbiota within the same comparative framework, this study aimed to separate tissue-associated microbial selection from rearing-environment-associated effects. This paired gut–gill design allowed us to evaluate whether two host mucosal niches with different levels of environmental exposure respond similarly or differently to the same contrast between conventional marine culture and saline–alkaline land-based culture.

## 2. Materials and Methods

### 2.1. Sample Collection and Methodology

Samples were collected from two contrasting aquaculture systems for *L. crocea*, including a marine culture system in Ningde and an indoor saline–alkaline culture system in Ningxia, China (Supplementary Table S1). Juvenile *L. crocea* with an initial body length of approximately 5–7 cm and an age of approximately three months were divided into the two rearing systems. Fish in both systems were maintained in culture tanks with a diameter of 6 m and a water depth of 2 m. The stocking density was approximately 100–150 fish per m^3^. Temperature, photoperiod, and feed conditions were maintained as consistently as possible between the two systems. Fish were fed commercial pellet feed twice daily at approximately 3–5% of body weight. During rearing, a small amount of water lost during siphoning was replenished, and aeration was supplied using nanotube aeration.

Sampling was conducted in the 14th month of the experiment. Samples were collected from two contrasting aquaculture systems for *L. crocea*, including a marine culture system in Ningde and a saline–alkaline culture system in Ningxia, China (Supplementary Table S1). A total of nine apparently healthy *L. crocea* individuals were collected from each rearing region. In each region, fish were sampled from three independent culture units, including ponds or cages, with three individuals collected from each culture unit. The sampled fish were at a comparable developmental stage, with an average body weight of approximately 164.34 g and an average body length of approximately 22.68 cm. All fish were fed Seahorse Brand Large Yellow Croaker Formula Feed, containing crude fat ≥ 6.0%, crude protein ≥ 45.0%, lysine ≥ 2.8%, crude ash ≤ 14.0%, crude fiber ≤ 5.0%, calcium 1.0–3.0%, total phosphorus ≥ 1.0%, sodium chloride 0.5–3.0%, and moisture ≤ 10.0%. No obvious external lesions, abnormal behavior, or disease symptoms were observed at the time of sampling. Available individual measurements of final body weight, total length, and body length at the time of microbiome sampling were compiled for fish from the marine and saline–alkaline rearing systems and are provided descriptively in Supplementary Table S1. Because these measurements were not collected under a study design intended for zootechnical performance comparisons at the independent culture-unit level, no inferential statistical comparison between the two rearing systems was conducted.

For host-associated samples, gut and gill tissues were collected from the same fish individuals. To obtain a representative culture-unit-level microbial profile while reducing the influence of extreme inter-individual variation, tissues from three individuals within each independent culture unit were pooled to generate one composite biological sample for DNA extraction. Therefore, the independent culture unit, rather than each individual fish, was treated as the biological replicate. Each region contained three independent composite samples for gut microbiota and three independent composite samples for gill microbiota. This pooling strategy was used to focus on broad microbial differences associated with host mucosal niche and rearing environment, although it does not allow the evaluation of individual-level consistency within each culture unit. The final sampling groups included control water from fish-free systems in an marine culture system (ND_CK_W) and saline–alkaline culture system (NX_CK_W), aquaculture water from stocked farming systems in marine culture (ND_W) and saline–alkaline culture (NX_W), gut samples from fish reared in a marine culture system and saline–alkaline culture (ND_I and NX_I), and gill samples from fish reared in a marine culture system and saline–alkaline culture (ND_G and NX_G). Fish-free control water refers to water maintained in fish-free culture units prepared with the same regional source water and managed under the same basic water-treatment conditions as the corresponding stocked aquaculture units, but without *L. crocea*. Therefore, ND_CK_W and NX_CK_W represent fish-free system water rather than natural ambient water bodies. For consistency across the manuscript and figures, sample names were standardized according to group identity and replicate number. Thus, individual sample labels were expressed as ND_CK_W1–3, NX_CK_W1–3, ND_W1–3, NX_W1–3, ND_I1–3, NX_I1–3, ND_G1–3, and NX_G1–3.

*L. crocea* individuals were randomly collected from the two aquaculture systems and processed aseptically. Fish were euthanized before tissue collection following institutional animal welfare guidelines, and gill and gut tissues were collected immediately after euthanasia to minimize post-mortem microbial changes. Before dissection, the external body surface was rinsed with sterile water and disinfected with 75% ethanol. Sterile scissors and forceps were used for tissue collection. Gill tissues were excised first using sterile instruments, and gut samples were collected after aseptic dissection. Instruments were sterilized or replaced between individuals to avoid cross-contamination. Tissue samples were transferred into sterile cryovials immediately after collection and placed in liquid nitrogen within 2 min. Water samples from both fish-free control systems and stocked aquaculture systems were collected simultaneously from the corresponding culture units. All samples were transferred into sterile containers, preserved on ice or in liquid nitrogen during field handling, transported under cold-chain conditions, and stored at −80 °C until DNA extraction.

### 2.2. Measurement of Environmental Variables

To characterize environmental heterogeneity between the marine system and the saline–alkaline system, water physicochemical parameters were measured during sampling for both fish-free control water and stocked aquaculture water. The measured variables included temperature, pH, salinity, dissolved oxygen, alkalinity, NO_2_^−^, NO_3_^−^, NH_4_^+^, PO_4_^3−^, total phosphorus, and other routine water quality indicators where available. Detailed values for each water sample are provided in Supplementary Table S1. These variables were used for correlation analysis and multivariate analyses to evaluate associations between water physicochemical conditions and microbial community composition.

### 2.3. Total Genomic DNA Extraction and Quality Inspection

Microbial genomic DNA was extracted from gut and gill samples using a DNA extraction kit according to the manufacturer’s instructions. Briefly, gut and gill tissues were aseptically collected and homogenized prior to DNA extraction. Then, 2 L of sample water was filtered through a filter membrane, and the DNA was extracted from the collected samples. The genomic DNA was isolated using DNeasy PowerSoil Pro Kit, following the manufacturer’s protocol (QIAGEN, GmbH, Hilden, Germany). The concentration of the extracted DNA was measured using a Qubit 3.0 Fluorometer (Life Technologies, ThermoFisher Scientific, Waltham, MA, USA) and the Equalbit 1x dsDNA HS Assay Kit (Vazyme, Nanjing, China, www.vazyme.com, accessed on 16 July 2026). Prior to sequencing, the quality and concentration of the DNA were determined again by 1.0% agarose gel electrophoresis and a NanoDrop^®^ ND-2000 spectrophotometer (Thermo Scientific Co., Ltd., Waltham, MA, USA).

### 2.4. Amplification of the 16S rRNA Gene

The primer sets 515FmodF (5′-GTGYCAGCMGCCGCGGTAA-3′) and 806R (5′-GGACTACHVGGGTWTCTAAT-3′) were used to amplify the V4 region of prokaryotic 16S rRNA genes, covering bacterial and archaeal lineages, where amplified, generating an amplicon length that could be reliably overlapped using Illumina MiSeq PE300 paired-end sequencing. Although the V3–V4 region can provide a longer taxonomic signal, the V4 region was considered appropriate for this study because it ensured stable read merging, reduced quality loss at read ends, and allowed comparison with many aquatic microbiome datasets generated using the same primer region. PCR was conducted at a 20 μL reaction volume, including 10 ng of DNA, 0.8 μL of each primer (10 μM), 4 μL 5× FastPfu buffer, 0.4 μL FastPfu polymerase, 0.2 μL BSA, and 250 μM dNTPs. PCR cycling conditions were as follows: initial denaturation at 95 °C for 3 min, followed by 29 cycles of denaturation at 95 °C for 30 s, annealing at 55 °C for 30 s, extension at 72 °C for 45 s, and a final extension at 72 °C for 10 min.

### 2.5. Illumina Sequencing and Bioinformatic Analysis

Purified amplicons were combined in equimolar proportions and sequenced using an Illumina MiSeq PE300 platform, following the standard protocols provided by Majorbio Bio-Pharm Technology Co., Ltd. (Shanghai, China).

The QIIME 2 pipeline was utilized for processing raw reads and downstream analysis [13]. In brief, raw sequence data were demultiplexed and primer-trimmed, followed by quality filtering, denoising, merging, and chimera removal using the DADA2 plugin [14,15]. Non-singleton amplicon sequence variants (ASVs) were aligned with MAFFT and used to construct a phylogeny with FastTree2 [16]. Alpha diversity metrics, including Chao1, Sobs, Shannon, and Coverage indices, were calculated to evaluate within-sample microbial richness and diversity. Beta diversity was assessed based on Bray-Curtis dissimilarity calculated from ASV abundance profiles, and NMDS ordination was used to visualize differences in microbial community structure among groups [17]. Taxonomy assignment was performed using the classify-sklearn naïve Bayes taxonomy classifier against the SILVA Release 138 Database (bacteria and archaea). After taxonomic assignment, ASVs assigned to chloroplasts, mitochondria, eukaryotes, or other non-target lineages were removed from the feature table before downstream analysis. Because archaeal ASVs accounted for a minor proportion of the dataset, the Results and Discussion mainly focus on bacterial community patterns. The resulting filtered ASV abundance table was used for all subsequent analyses, including alpha diversity, beta diversity, taxonomic composition, differential abundance analysis, environmental correlation analysis, heatmap visualization, and co-occurrence network analysis.

### 2.6. Statistical Analysis and Visualization

All statistical analyses were performed using the filtered ASV abundance table after removing chloroplast, mitochondrial, eukaryotic, and other non-target sequences. Alpha diversity indices, including Chao1, Sobs, Shannon, and Coverage, were used to evaluate within-sample microbial richness, diversity, and sequencing coverage. Differences in alpha diversity among groups were tested using the Kruskal–Wallis test. When significant differences were detected, pairwise comparisons were performed using Wilcoxon rank-sum tests. Beta diversity was assessed using Bray–Curtis dissimilarity calculated from ASV relative abundance profiles. NMDS was used to visualize differences in microbial community structure among groups. Group-level differences in community composition were tested using ANOSIM with 999 permutations. Taxonomic composition was summarized at the phylum, class, order, family, and genus levels based on relative abundance. Shared and unique taxa among groups were visualized using Venn diagrams at different taxonomic levels. Differentially abundant genera among water, gut, and gill groups were identified using the Kruskal–Wallis test followed by pairwise Wilcoxon rank-sum tests. To further evaluate the relationships between microbial community composition and environmental variables, db-RDA was performed based on Bray–Curtis dissimilarity using the measured physicochemical variables as explanatory factors. Spearman correlation analysis was performed to evaluate associations between dominant genera and environmental variables. Correlations with adjusted *p* < 0.05 were considered statistically significant unless otherwise stated. Heatmaps were generated based on the relative abundance profiles of dominant ASVs or genera after appropriate normalization. Correlation-based topological analysis was performed using dominant taxa retained after prevalence and abundance filtering. Pairwise Spearman correlations were calculated among taxa, and only significant associations meeting the predefined thresholds of |r| > 0.6 and adjusted *p* < 0.05 were retained for subsequent topological role assessment. Structural keystoneness was calculated at the genus level to identify taxa with relatively high topological importance, and the complete genus-level results are provided in Supplementary Table S4. Zi–Pi values were calculated to classify the topological roles of ASV nodes, and the complete classification results are provided in Supplementary Table S4. Because the analysis was based on compositional 16S rRNA gene amplicon data and pairwise statistical associations, the observed patterns were interpreted as correlation-based topological summaries rather than direct ecological interactions or a fully resolved node–edge microbial network. To provide an exploratory assessment of potential microbial functional profiles, PICRUSt2 was used to predict functional gene content based on the filtered ASV abundance table. ASV representative sequences were placed into a reference phylogeny, and predicted gene family abundances were inferred and normalized according to the PICRUSt2 workflow. Predicted Kyoto Encyclopedia of Genes and Genomes Orthology (KO) profiles were summarized across samples, and the dominant predicted KO categories were visualized using heatmaps. Statistical analyses and visualizations were conducted in R using packages including vegan, ggplot2, pheatmap or ComplexHeatmap, psych or Hmisc, and igraph. Statistical significance was defined as *p* < 0.05.

### 2.7. Nucleotide Accession Numbers

The raw sequence data are available in the Genome Sequence Archive (Genomics, Proteomics & Bioinformatics 2021) at the National Genomics Data Center (Nucleic Acids Res 2022), China National Center for Bioinformation/Beijing Institute of Genomics, Chinese Academy of Sciences (accession codes: CRA040277), and can be accessed at https://ngdc.cncb.ac.cn/gsa (accessed on 16 July 2026). Data availability for peer review: CRA040277 (https://ngdc.cncb.ac.cn/gsa/search?searchTerm=CRA043344, accessed on 16 July 2026), the data has been released.

## 3. Results

### 3.1. Alpha Diversity of the Microbial Communities

**Figure 1:**
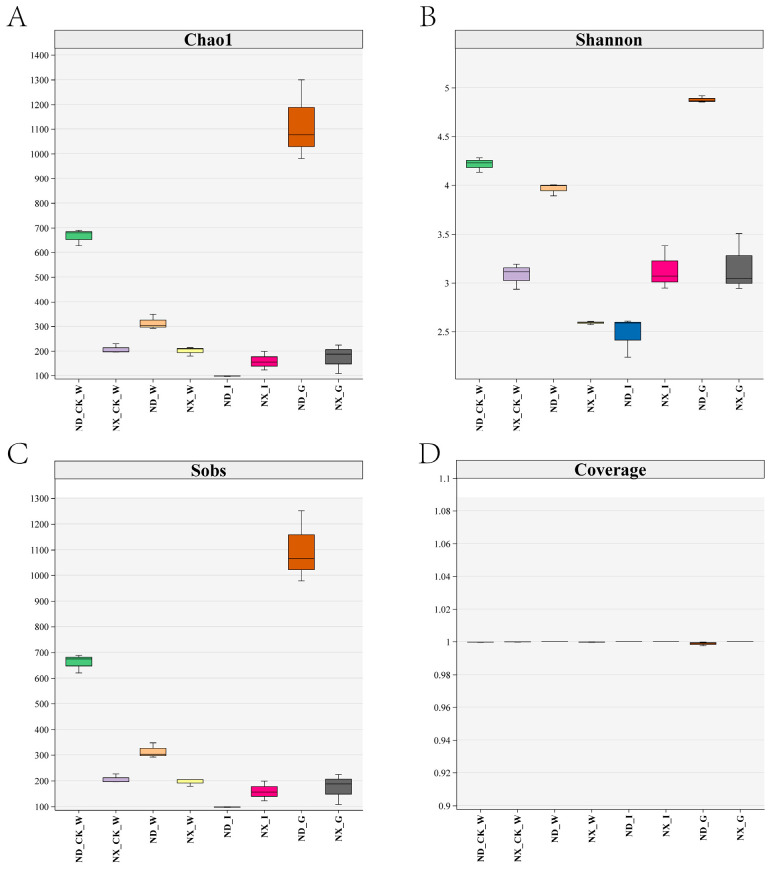
Alpha diversity estimators for microorganisms: (**A**) Chao1 for bacteria, (**B**) Shannon for bacteria, (**C**) Sobs for bacteria, (**D**) Coverage for bacteria.

Basic zootechnical characteristics of the fish at the time of microbiome sampling, including final body weight, total length, and body length, are provided descriptively in Supplementary Table S1. Alpha diversity was evaluated using the Chao1, Coverage, Shannon, and Sobs indices to compare microbial diversity among the different sample groups (Figure 1). Overall, clear differences were observed between water samples and fish-associated samples. The control and aquaculture water samples generally exhibited higher richness and diversity, whereas gut and gill samples showed relatively lower alpha diversity, indicating that host-associated microbial communities were more selective and less diverse than those in the surrounding water (Figure 1). In addition, differences between the marine culture and saline–alkaline culture system were also evident in fish-associated samples, suggesting that aquaculture conditions influenced the diversity of host-associated microorganisms.

### 3.2. NMDS Analysis of Microbial Community Structure

**Figure 2:**
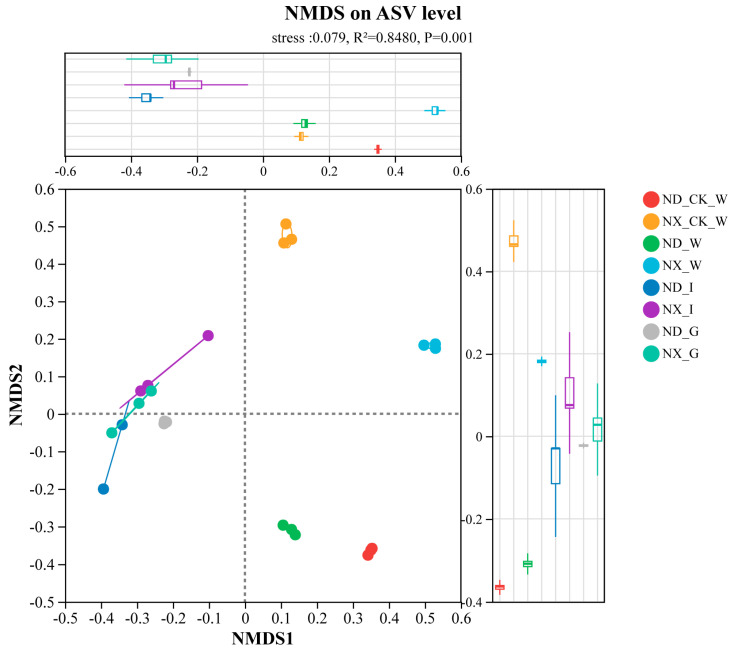
NMDS ordination of microbial community structure based on Bray–Curtis dissimilarity calculated from the filtered ASV abundance table. Group-level differences were tested using ANOSIM with 999 permutations. Colors indicate sample groups.

An integrated NMDS analysis based on Bray–Curtis dissimilarity of ASV abundance profiles was performed using all water, gut, and gill samples from the two rearing systems (Figure 2). The ordination showed clear separation among the eight sample groups, including fish-free control water and aquaculture water, gut, and gill samples from the marine and saline–alkaline culture systems. Water samples were clearly separated from host-associated samples, indicating that gut and gill microbial communities were distinct from the surrounding water communities. In addition, gut and gill samples occupied different ordination spaces, suggesting host mucosal niche-associated differentiation. Samples from the marine culture system and saline–alkaline culture system were also separated within the same sample type, indicating that rearing environment further contributed to microbial community variation. The integrated NMDS analysis showed a low stress value of 0.079, and the group-level difference was significant (R^2^ = 0.8480, *p* = 0.001). These results indicate that microbial community structure was jointly associated with host mucosal niche and rearing environment.

### 3.3. Microbial Community Composition Across Different Taxonomic Levels

**Figure 3:**
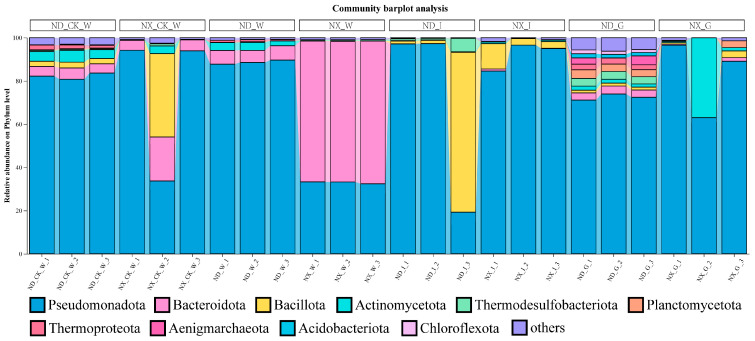
Microbial community composition at phylum level.

At the phylum level, the microbial communities across all samples were predominantly composed of *Pseudomonadota*, followed by *Bacteroidota*, *Bacillota* and *Actinomycetota*, although their relative abundances varied markedly among habitats and regions (Figure 3). Water samples from both the marine culture and saline–alkaline culture system were mainly dominated by *Pseudomonadota*, whereas fish-associated samples, especially gut samples, showed increased contributions of *Bacteroidota*, *Bacillota*, and *Actinomycetota*. Gill samples, particularly those from the saline–alkaline culture system, also exhibited a relatively higher proportion of *Actinomycetota*, indicating clear habitat- and region-dependent compositional shifts.

At the class level, *Alphaproteobacteria* and *Gammaproteobacteria* were dominant in most water samples, whereas gut and gill samples were characterized by more heterogeneous assemblages (Supplementary Figure S1). Gut samples contained higher proportions of *Bacteroidia*, *Bacilli*, and *Clostridia*, while gill-associated microorganisms were enriched in *Alphaproteobacteria* and *Gammaproteobacteria*. The taxonomic shifts were more evident in Ningxia fish-associated samples, especially in the gut, suggesting that distinct microbial community patterns were associated with saline–alkaline aquaculture conditions.

At the order and family levels, clear differences were also observed among habitats. Water samples were mainly represented by *Rhodobacterales*, *Pseudomonadales*, *Hyphomicrobiales*, and *Burkholderiales*, whereas host-associated samples were enriched in *Flavobacteriales*, *Mycoplasmatales*, *Lactobacillales*, *Enterobacterales*, and *Sphingomonadales* (Supplementary Figure S2). At the family level, *Paracoccaceae*, *Beijerinckiaceae*, *Moraxellaceae*, *Comamonadaceae*, and *Flavobacteriaceae* were among the dominant taxa, but their relative abundances differed substantially across sample types (Supplementary Figure S3). Gut samples from the marine culture and saline–alkaline culture system exhibited distinct family-level profiles, and similar regional divergence was also observed in gill samples.

**Figure 4:**
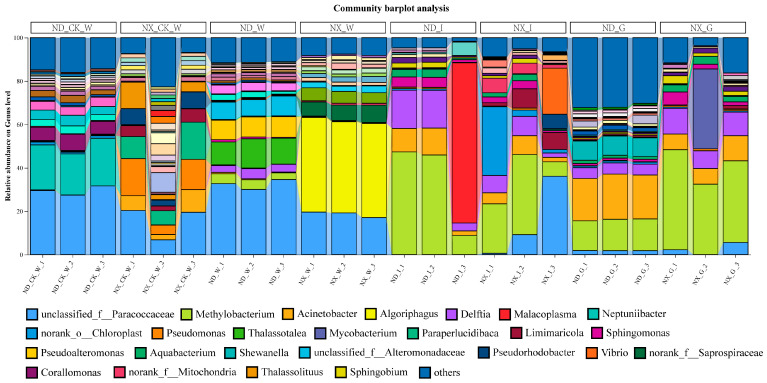
Microbial community composition at genus level.

At the genus level, compositional differences became more pronounced. Water samples were mainly dominated by *unclassified_f_Paracoccaceae*, *Methylobacterium*, *Acinetobacter*, *Algoriphagus*, and *Delftia*, whereas fish-associated samples were enriched in genera such as *Malacoplasma*, *Pseudomonas*, *Sphingomonas*, *Shewanella*, *Vibrio*, and *Photobacterium*, together with several unclassified bacterial groups (Figure 4). Gut microbiota from the marine culture and saline–alkaline culture system formed clearly distinct assemblages. Gill communities also differed between the two regions, although they retained partial similarity to the rearing water microbial community. Overall, these results indicate that microbial community composition differed markedly among host mucosal niches and between the two contrasting rearing systems across multiple taxonomic levels.

### 3.4. Shared and Unique Taxa Revealed by Venn Analysis

Venn analysis was used to compare the shared and unique taxa among groups at the phylum, family, genus, and ASV levels (Supplementary Figure S4). Among the four water groups, a considerable number of taxa were shared across samples, but each group also contained unique lineages, and the number of unique taxa increased markedly from higher to lower taxonomic levels. At the phylum level, 17 phyla were shared among the four water groups, whereas the number of shared taxa decreased at the family and genus levels, and only a limited fraction of ASVs was shared across all water samples. Meanwhile, each water group retained a substantial number of unique ASVs, especially the marine control water group.

A similar pattern was observed in host-associated samples (Supplementary Figure S5). In the gut samples, only a small proportion of taxa were shared between the two culture systems at the ASV, genus, family, and phylum levels, while each system retained many unique taxa, particularly at the ASV and genus levels. Saline–alkaline culture gut samples contained more unique ASVs and genera than marine culture gut samples, indicating stronger regional divergence in gut microbial composition.

For the gill samples, shared taxa between the two culture systems were also limited at lower taxonomic levels (Supplementary Figure S6). The number of shared phyla remained relatively high, but overlap decreased markedly at the family, genus, and ASV levels. Marine culture gill samples contained more unique ASVs and genera than saline–alkaline culture gill samples, suggesting that regional effects on gill-associated microorganisms were also substantial. Overall, the Venn analysis indicates that microbial divergence between the marine culture and saline–alkaline culture became progressively more pronounced at a finer taxonomic resolution.

### 3.5. Differentially Enriched Taxa Among Sample Groups

**Figure 5:**
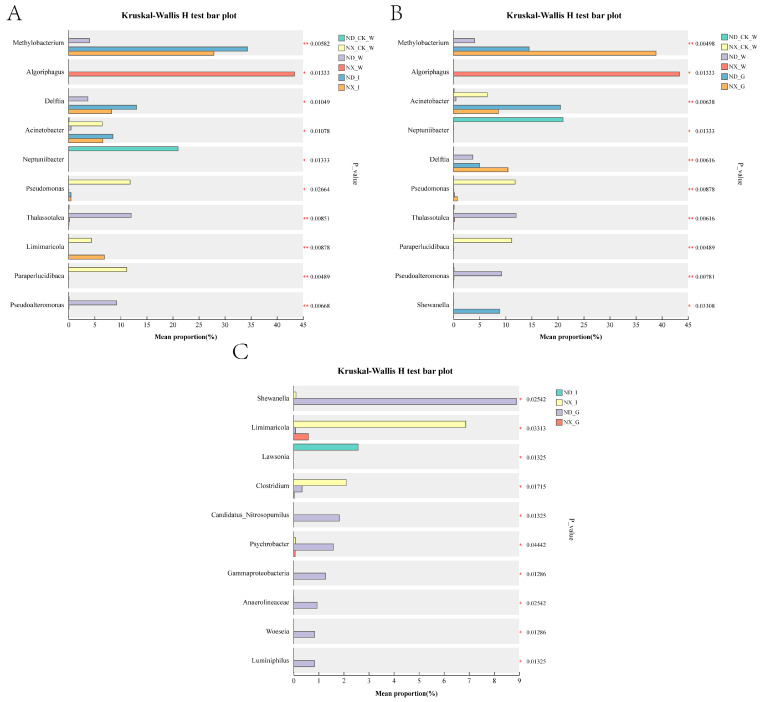
Differentially enriched genera among water, gut, and gill samples. (**A**) Differentially enriched genera between gut samples and water groups. (**B**) Differentially enriched genera between gill samples and water groups. (**C**) Differentially enriched genera between gut and gill samples. Significant differences were tested using the Kruskal–Wallis test followed by pairwise Wilcoxon rank-sum tests. * *p* < 0.05, ** *p* < 0.01.

To further identify taxa associated with host mucosal niche and water environments after sequence filtering, dominant bacterial genera were compared among gut samples, gill samples, and the corresponding water groups. In the comparison between gut samples and water groups, *Methylobacterium*, *Algoriphagus*, *Delftia*, *Acinetobacter*, *Neptuniibacter*, *Pseudomonas*, *Thalassotalea*, *Paraperlucidibaca*, and *Limimaricola* showed significant differences among groups. Among these taxa, *Methylobacterium*, *Delftia*, and *Acinetobacter* were more abundant in gut samples, whereas *Algoriphagus*, *Neptuniibacter*, *Pseudomonas*, *Thalassotalea*, and *Paraperlucidibaca* were mainly enriched in water samples (Figure 5A).

For the gill-associated microbiota, *Methylobacterium*, *Algoriphagus*, *Acinetobacter*, *Neptuniibacter*, *Delftia*, *Pseudomonas*, *Thalassotalea*, *Paraperlucidibaca*, *Pseudoalteromonas*, and *Shewanella* differed significantly among gill and water groups. *Shewanella* was more strongly associated with gill samples, whereas *Pseudoalteromonas*, *Thalassotalea*, *Paraperlucidibaca*, and *Pseudomonas* were more characteristic of the surrounding water communities. In contrast, *Methylobacterium* and *Acinetobacter* contributed more strongly to fish-associated samples (Figure 5B).

Further comparison between gut and gill microbiota identified several bacterial taxa that distinguished the two host mucosal niches, including *Shewanella*, *Limimaricola*, *Lawsonia*, *Clostridium*, *Candidatus_Nitrosopumilus*, *Psychrobacter*, *norank_Gammaproteobacteria*, and *norank_Anaerolineaceae*. Among these, *Shewanella* and *Psychrobacter* were more closely associated with gill samples, whereas *Clostridium* and *Lawsonia* showed relatively higher abundance in gut samples, reflecting tissue-associated microbial differentiation within the host (Figure 5C).

### 3.6. Heatmap Analysis of Dominant ASVs

**Figure 6:**
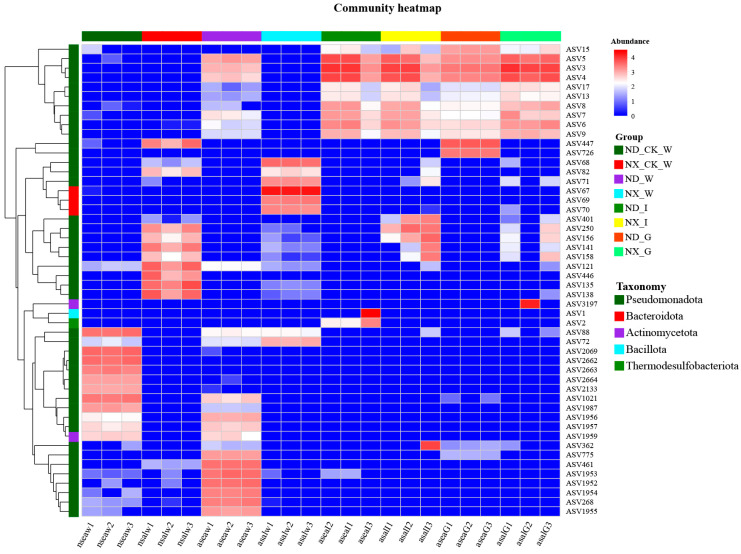
Relative abundance and taxonomic classification of top 50 ASVs.

To further characterize fine-scale compositional differences among sample groups, a heatmap based on the top 50 ASVs was constructed (Figure 6). The results showed clear variation in ASV distribution among water, gut, and gill samples, with samples from the same habitat generally clustering together. Water samples from the marine culture and saline–alkaline culture system were mainly enriched in a shared set of dominant ASVs, whereas fish-associated samples displayed more distinct and habitat-specific ASV patterns. Gut samples from the marine and saline–alkaline system formed separate clusters and were characterized by different dominant ASV assemblages. Gill samples were also clearly separated from water and gut samples, although they retained partial similarity to the surrounding rearing water. The dominant ASVs were mainly affiliated with *Pseudomonadota*, *Bacteroidota*, *Bacillota*, *Actinomycetota*, and *Thermodesulfobacteriota*, although their relative contributions varied substantially among groups.

### 3.7. Correlations Between Environmental Factors and Microbial Community Structure

**Figure 7:**
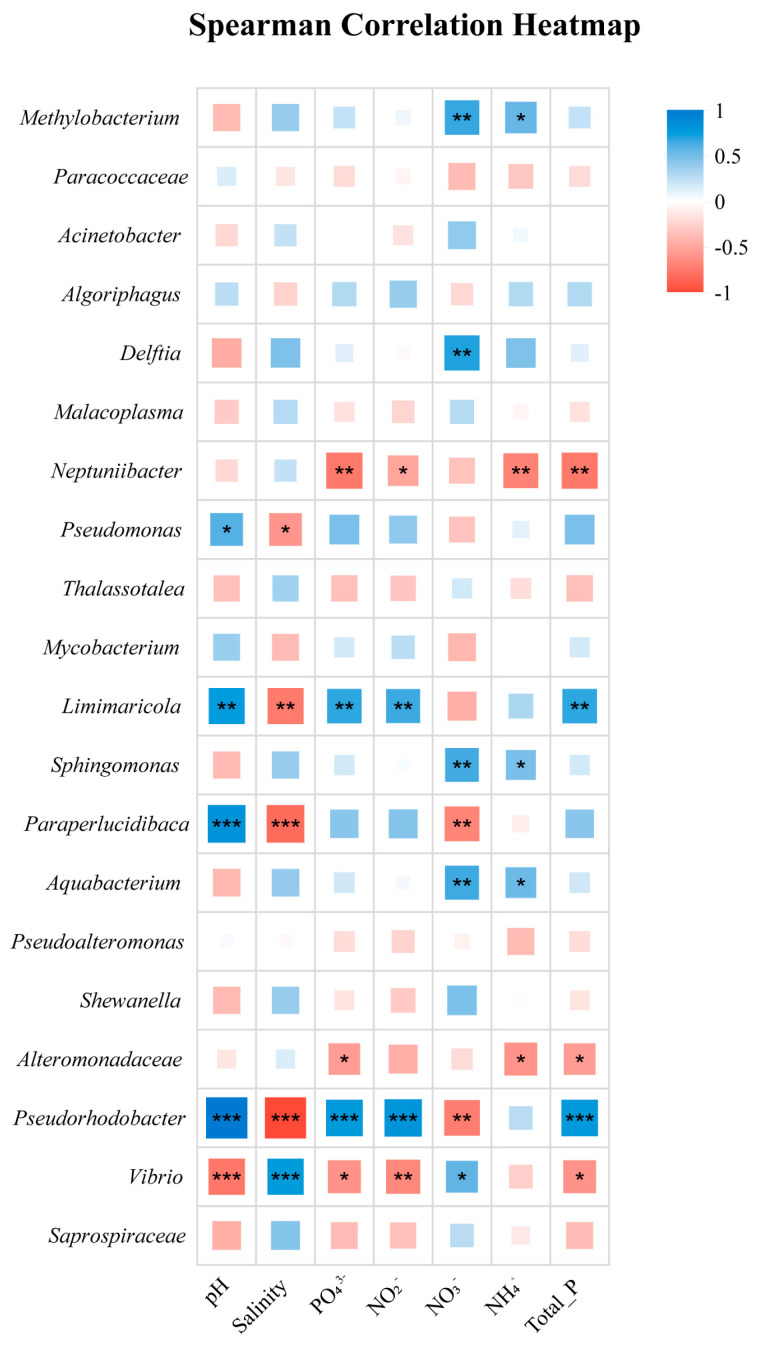
Spearman correlations between dominant genera and measured environmental variables. Correlations were calculated using the filtered abundance table. Positive and negative correlations are indicated by color intensity. * *p* < 0.05, ** *p* < 0.01, *** *p* < 0.001.

Spearman correlation analysis was performed to explore the relationships between environmental variables and dominant genera (Supplementary Table S2). The results showed that several taxa were significantly associated with water physicochemical factors, suggesting that environmental heterogeneity was linked to variation in microbial community composition. Salinity, NO_3_^−^, NH_4_^+^, and total phosphorus were among the major factors correlated with dominant taxa (Figure 7).

Among the dominant genera, *Methylobacterium*, *Delftia*, *Sphingomonas*, and *Aquabacterium* showed significant positive correlations with NO_3_^−^, whereas *Neptuniibacter* exhibited significant negative correlations with PO_4_^3−^, NH_4_^+^, and Total_P. Pseudomonas was positively correlated with pH, PO_4_^3−^, and Total_P, but negatively correlated with salinity. Similarly, *Paraperlucidibaca* and *Limimaricola* were positively associated with pH, PO_4_^3−^, NO_2_^−^, and Total_P, whereas both showed negative correlations with salinity. *Pseudorhodobacter* displayed positive correlations with pH, PO_4_^3−^, NO_2_^−^, and Total_P, but negative correlations with salinity and NO_3_^−^. In contrast, *Vibrio* was positively correlated with salinity and NO_3_^−^ and negatively associated with pH, PO_4_^−^, NO_2_^−^, and Total_P.

The correlations between dominant genera and salinity or nutrient-related variables further suggest that environmental effects were not expressed uniformly across all taxa. Instead, different bacterial groups showed distinct associations with physicochemical gradients, indicating that the rearing environment may influence microbial assembly by modifying both the environmental species pool and the selective conditions encountered by host-associated communities. In this sense, salinity and nutrient availability should be interpreted not simply as background descriptors but as potential ecological filters associated with the divergence between the marine and saline–alkaline systems. However, because this study was observational, these associations should be considered hypothesis-generating rather than direct evidence of causality.

**Figure 8:**
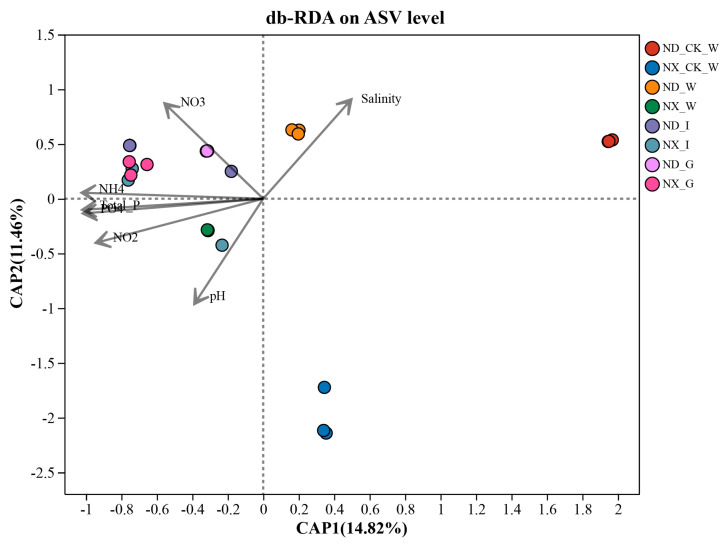
Distance-based redundancy analysis (db-RDA) showing associations between microbial community structure and measured environmental variables at the ASV level.

To further assess the associations between microbial community structure and measured environmental variables, db-RDA was performed based on Bray–Curtis dissimilarity using the filtered ASV abundance table (Figure 8). The first two constrained axes, CAP1 and CAP2, explained 14.82% and 11.46% of the microbial community variation, respectively, together accounting for 26.28% of the variation. The db-RDA ordination showed clear separation among several water and host-associated sample groups, indicating that microbial community structure was associated with measured physicochemical gradients.

Envfit analysis further showed that all measured environmental variables were significantly associated with microbial community structure (Supplementary Table S1). NO_3_^−^ showed the strongest association with community variation (r^2^ = 0.8956, *p* = 0.001), followed by NH_4_^+^ (r^2^ = 0.6399, *p* = 0.001), salinity (r^2^ = 0.5253, *p* = 0.001), and pH (r^2^ = 0.4628, *p* = 0.001). PO_4_^3−^ (r^2^ = 0.3212, *p* = 0.014), NO_2_^−^ (r^2^ = 0.2825, *p* = 0.035), and total phosphorus (r^2^ = 0.2766, *p* = 0.037) were also significantly associated with microbial community structure.

In the db-RDA ordination, salinity was mainly aligned with the positive direction of CAP1 and CAP2, whereas pH was mainly associated with the negative direction of CAP2 (Figure 8). NO_3_^−^ was oriented toward the negative direction of CAP1 and the positive direction of CAP2, while NH_4_^+^, PO_4_^3−^, NO_2_^−^, and total phosphorus were mainly associated with the negative direction of CAP1. These results suggest that microbial community variation was associated with multiple physicochemical gradients rather than a single environmental factor. Therefore, the marine culture and saline–alkaline culture system microbial divergence should be interpreted as a rearing-system-associated pattern linked to combined environmental variation rather than as direct evidence of salinity or alkalinity alone driving community differences.

### 3.8. Topological Role Analysis Based on Significant Microbial Associations

**Figure 9:**
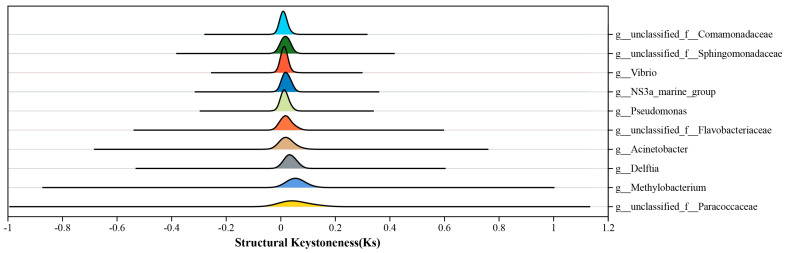
Structural keystoneness of dominant genera based on correlation-derived topological analysis. Structural keystoneness values were calculated to identify taxa with relatively high topological importance. Genus names are italicized where taxonomic assignment reached the genus level, whereas unclassified or non-ranked taxa are shown in roman type.

To identify taxa with relatively high topological importance, correlation-based topological role analysis was performed using significant Spearman associations among microbial taxa. The complete structural keystoneness results and Zi–Pi classification results are provided in Supplementary Tables S3 and S4, respectively. At the genus level, 50 taxa were included in the structural keystoneness analysis (Figure 9). The highest median structural keystoneness was observed for *unclassified_f_Paracoccaceae*, followed by *Methylobacterium*, *Delftia*, *Acinetobacter*, *unclassified_f_Flavobacteriaceae*, *Pseudomonas*, NS3a_marine_group, *Vibrio*, *unclassified_f_Sphingomonadaceae*, and *unclassified_f_Comamonadaceae*. These taxa showed relatively high topological importance in the correlation-based association structure.

**Figure 10:**
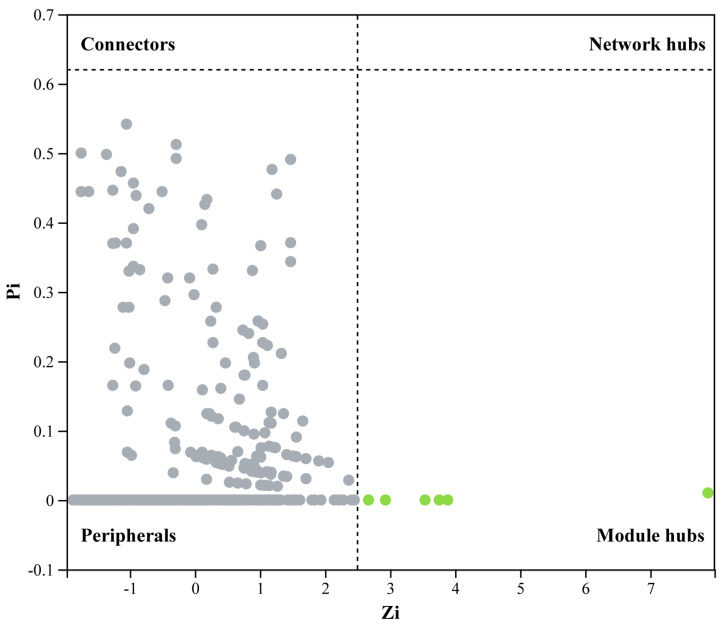
Zi–Pi plot showing the topological roles of ASV nodes based on significant microbial associations. Nodes were classified according to within-module connectivity (Zi) and among-module connectivity (Pi). The plot was used to identify peripheral nodes, module hubs, connectors, and network hubs.

The Zi–Pi analysis classified 4263 ASV nodes into different topological roles (Figure 10). Most nodes were classified as peripherals, accounting for 4257 nodes, whereas only 6 nodes were identified as module hubs. No connectors or network hubs were detected. This pattern indicates that most microbial nodes mainly contributed to local within-module connectivity, while only a small number of nodes showed high within-module connectivity. Because these results were derived from compositional 16S rRNA gene amplicon data and pairwise statistical associations, they should be interpreted as correlation-based topological summaries rather than evidence of direct microbial interactions.

Overall, these results indicate that although most taxa occupied peripheral positions in the inferred co-occurrence network, several dominant genera showed relatively high network topological importance. However, because the network was inferred from compositional 16S rRNA gene amplicon data, these patterns should be interpreted as statistical co-occurrence-based indications rather than direct evidence of ecological interactions, functional roles, or causal relationships.

### 3.9. Predicted Functional Profiles Based on PICRUSt2 Analysis

**Figure 11:**
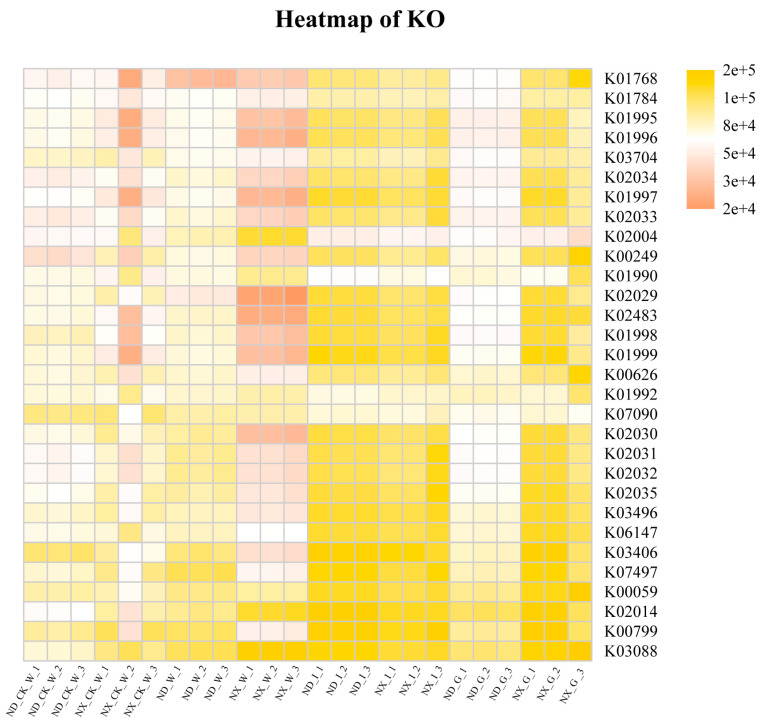
Heatmap of dominant predicted KO profiles inferred by PICRUSt2. Samples are grouped according to rearing system and sample type in the following order: marine fish-free control water (ND_CK_W), saline–alkaline fish-free control water (NX_CK_W), marine aquaculture water (ND_W), saline–alkaline aquaculture water (NX_W), marine gut samples (ND_I), saline–alkaline gut samples (NX_I), marine gill samples (ND_G), and saline–alkaline gill samples (NX_G).

To further explore the potential functional differences among water, gut, and gill microbial communities, PICRUSt2-based functional prediction was performed using the filtered ASV abundance table. The heatmap of dominant predicted KO profiles showed clear functional differences among sample types and between the two rearing systems (Figure 11). Overall, host-associated samples, especially gut samples, exhibited higher predicted abundances across multiple KO categories than several water samples. This pattern suggests that the gut microbiota may harbor a more distinct and concentrated predicted functional potential compared with the surrounding water microbial community.

The predicted KO profiles also differed between the marine culture and saline–alkaline culture system. Gut samples from both systems showed relatively high predicted KO abundances, whereas gill samples displayed more variable patterns. In particular, several saline–alkaline culture system gill samples showed relatively high predicted abundances for multiple KO categories, suggesting that gill-associated functional potential may be linked to rearing-system-associated microbial differences. Water samples also showed distinct predicted functional patterns, indicating that environmental microbial reservoirs may differ not only in taxonomic composition but also in predicted functional profiles.

These results provide exploratory evidence that microbial communities associated with different host mucosal niches and rearing environments may differ in predicted functional potential. However, because these functions were inferred from 16S rRNA gene profiles, they should be interpreted cautiously as putative functional capacities rather than direct evidence of gene presence, expression, or metabolic activity.

## 4. Discussion

The present study showed that the gut and gill microbiota of large yellow croaker were associated with both host mucosal niche tissue specificity and contrasting rearing systems [1,2,3,8,18]. Although the surrounding water likely represented the primary external microbial source, the gut and gill communities were clearly differentiated from the water microbial community, indicating that host-associated assemblages were not passively derived from the environment but selectively shaped by host-related filtering processes [1,2,3,19,20]. Similar patterns have been widely reported in fish, where gut microbiota differ from ambient water communities [3,21,22] and gill-associated microorganisms also form a distinct mucosal assemblage influenced by host-specific factors [2,8,23,24]. This host–environment association has been increasingly recognized as an important pattern in fish microbiome assembly [20,25,26].

A major finding of this study is the pronounced divergence between the marine culture and saline–alkaline culture system, especially in host-associated samples. This result suggests that microbial assembly in the large yellow croaker is highly sensitive to local farming context and environmental background [8,19,25,27]. Such marine and saline–alkaline differentiation is ecologically meaningful because broad phylum-level patterns can remain partly similar while strong divergence emerges at lower taxonomic levels, where habitat filtering and host selection become more evident [24,27,28,29]. Previous studies in fish have similarly shown that rearing environment, habitat, and farming mode can reshape host-associated microbial communities without necessarily causing complete turnover at higher taxonomic ranks [19,27,29,30]. In large yellow croaker specifically, gut bacterial communities were previously shown to differ from both feed and culture water, further supporting the view that local culture conditions exert substantial effects on host-associated microorganisms [3].

The stronger regional separation observed in the intestine than in the gill is also biologically plausible. The intestine is more strongly constrained by digestion, nutrient utilization, and immune regulation and, therefore, usually exhibits stronger niche selectivity than external mucosal tissues [21,26,31]. By contrast, the gill is continuously exposed to the surrounding water column and therefore tends to retain a greater environmental signal [2,8,23,24,32]. This interpretation agrees with previous studies showing that gill microbiota are distinct from gut microbiota but remain more responsive to environmental change than gut communities [2,23,24,33]. A recent study on large yellow croaker further demonstrated that the microorganisms associated with skin, gill, and gut differ among tissues and across farming conditions, reinforcing the idea that tissue-specific selection and environmental forcing act together rather than independently [8].

The stronger differentiation of the gut microbiota may be explained by the relatively enclosed and host-regulated nature of the intestinal mucosal environment. Compared with the surrounding water and the gill surface, the intestine is more directly shaped by dietary inputs, digestive processes, nutrient availability, mucus secretion, epithelial barriers, local immune regulation, and possibly lower oxygen availability [23]. These conditions can create a selective microenvironment that favors microbial taxa adapted to intestinal colonization and substrate utilization. Therefore, the clearer separation of gut samples between the marine and saline–alkaline culture systems may reflect not only environmental differences but also the interaction between external rearing conditions and stronger internal host filtering within the intestinal niche [8].

In contrast, the gill is a highly exposed mucosal surface that is continuously bathed by the rearing water [2]. In addition to its respiratory function, the fish gill is a major site of osmoregulation, ion regulation, acid-base balance, and nitrogenous waste excretion [34]. These physiological functions may partly explain why the gill microbiota retained stronger environmental signals than the intestine. Under saline–alkaline culture conditions, changes in salinity, alkalinity, pH, dissolved oxygen, and nutrient availability may directly influence the gill mucosal surface, including ion gradients, mucus properties, epithelial stress, and the local pool of microorganisms available for colonization [32]. The gill microbiota may therefore reflect a balance between host mucosal selection and continuous environmental microbial input. This interpretation is consistent with previous studies showing that fish gill microbiota are distinct from intestinal and environmental communities, but remain closely linked to external exposure and gill-specific physiological functions [24].

The reduced overlap from phylum to ASV level suggests that ecological divergence became progressively stronger at finer resolutions, which is a common feature in microbial community differentiation [19,24,27,28]. In aquaculture-associated fish microbiomes, comparable patterns have been reported when contrasting rearing environments or habitat types were examined, with lower taxonomic levels capturing environmental and host filtering more effectively than high-rank summaries [19,29]. This implies that the microbial distinction between the marine and saline–alkaline culture systems is true ecological restructuring rather than only broad compositional fluctuation [19,28].

The environmental correlations observed in this study provide a likely mechanistic explanation for this regional divergence. In particular, salinity and nutrient-related variables were strongly associated with several dominant taxa, suggesting that physicochemical heterogeneity contributed substantially to microbial assembly [25,30]. Salinity is one of the best-recognized ecological filters in aquatic microbial systems and has repeatedly been shown to alter fish-associated gut microbiota [22,30]. Zhao et al. demonstrated that salinity significantly affected the gut microbiota of farmed Chinook salmon [27], while studies in Atlantic salmon and tilapia have also shown that rearing system and water conditions can restructure gut microbial communities [29,30]. Reviews of fish microbiome ecology likewise emphasize that water chemistry, salinity, and nutrient availability are central drivers linking environmental microorganisms with fish-associated communities [21,25,26]. In the present study, the contrast between the marine culture system and the saline–alkaline culture system may reflect different environmental and management-related conditions that were associated with distinct microbial assemblages on both the environmental microbial reservoir and host mucosal niche, thereby contributing to the observed community divergence [25,29,30]. The db-RDA and Envfit analyses further supported the association between measured physicochemical variables and microbial community structure. Notably, NO_3_^−^ showed the strongest association with community variation, followed by NH_4_^+^, salinity, and pH, while PO_4_^3−^, NO_2_^−^, and total phosphorus were also significantly related to community structure. These results indicate that microbial community variation was associated with multiple environmental gradients rather than salinity alone. This finding directly addresses the potential confounding among environmental variables in the comparison between the marine culture system and the saline–alkaline culture system. Rather than attributing microbial divergence solely to saline–alkaline versus marine aquaculture conditions, the observed patterns should be interpreted as associations with a combination of measured physicochemical variables and unmeasured production system-related factors.

The differentially enriched taxa identified here should therefore be interpreted as niche-associated assemblages rather than simple taxonomic markers. Water-enriched taxa likely represent lineages favored by the surrounding physicochemical environment, whereas intestine- and gill-enriched taxa more likely reflect host selection, mucosal colonization capacity, and adaptation to tissue-specific conditions [1,2,24]. This interpretation is consistent with the “source-and-selection” framework increasingly used in fish microbiome studies, in which environmental communities provide the colonist pool, but only part of this pool is retained after host filtering [25,26]. In large yellow croaker, previous work has shown that host-associated gut communities differ from environmental and dietary microbial sources [3], and broader marine fish studies similarly support the idea that the host body site is a strong determinant of microbiome structure [28,33]. Several water-associated genera, such as *Pseudomonas*, *Thalassotalea*, *Paraperlucidibaca*, and *Pseudoalteromonas*, may reflect the influence of the surrounding aquatic microbial reservoir and local physicochemical conditions. In contrast, gut-associated genera such as *Clostridium* and *Lawsonia* may be linked to the selective conditions of the gut habitat, including nutrient availability, anaerobic microenvironments, and host immune filtering. Gill-associated genera such as *Shewanella* and *Psychrobacter* may reflect the combined influence of mucosal colonization and direct exposure to rearing water.

Another notable result is that only a limited number of taxa showed relatively high topological importance in the co-occurrence network. Most taxa occupied peripheral positions, suggesting that the network was mainly composed of locally connected members rather than broadly connected hubs. However, co-occurrence network analysis is inherently correlative and cannot directly demonstrate functional roles, causal interactions, or probiotic potential [5,26]. This pattern may have ecological implications, but the potential roles of these taxa in community persistence, resilience, or recovery cannot be confirmed without independent functional validation [5,25]. These taxa may provide useful candidates for future studies, but their ecological functions and potential applications in microbiome regulation require further validation through metagenomic analysis, cultivation-based assays, and host-associated experiments.

The PICRUSt2-based results provide a preliminary functional perspective for interpreting these taxonomic differences among water, gut, and gill microbial communities [35]. Because PICRUSt2 infers functional profiles from 16S rRNA gene sequences using reference-based prediction, these results should be regarded as putative functional potential rather than direct evidence of functional gene abundance, gene expression, or metabolic activity [35]. Nevertheless, predicted KO profiles can be useful for generating hypotheses about how microbial functional capacity may vary among host mucosal niches and rearing environments [25,26,35].

In the present study, the relatively stronger predicted functional profiles in gut samples may be associated with the more selective and nutrient-rich intestinal environment [22,27,32]. Gut-enriched taxa such as *Clostridium* and *Lawsonia* may be linked to microbial functions related to organic substrate utilization, nutrient transformation, and host-associated metabolism, although these roles require confirmation by metagenomic, metatranscriptomic, metabolomic, or cultivation-based approaches [21,26,31,35]. By contrast, the more variable predicted functional profiles in gill samples, particularly in some saline–alkaline culture samples, may reflect the direct influence of water chemistry on the gill mucosal microbiota [2,23,24,34]. Gill-associated taxa such as *Shewanella* and *Psychrobacter*, together with water-associated genera such as *Pseudomonas*, *Thalassotalea*, *Paraperlucidibaca*, and *Pseudoalteromonas*, may contribute to differences in predicted functional potential related to environmental adaptation, nutrient cycling, and microbial stress responses [32,34]. These interpretations remain hypothetical, but they provide a useful framework for future shotgun metagenomic, metatranscriptomic, metabolomic, or cultivation-based studies to validate functional differences between gut and gill microbiota under contrasting rearing environments [26,34].

From an applied perspective, the present findings suggest that microbiome-based management strategies for large yellow croaker should be culture-system-specific rather than universal [18,25]. The clear separation between marine culture and saline–alkaline culture indicates that microbial intervention strategies cannot be assumed to transfer directly between contrasting farming systems [25,30]. Instead, future microbiome-informed management, candidate taxa validation, and health monitoring should take both tissue specificity and rearing-system-associated differences into account [5,31]. This point is especially relevant for the development of “marine fish cultured on land” because altered salinity and water chemistry may reshape not only environmental microorganisms but also the host-associated communities that are closely linked to mucosal health, nutrient processing, and overall fish performance [27,31].

Overall, the present study suggests a two-layer ecological pattern of microbial assembly in large yellow croaker: host tissue was associated with clear niche differentiation, while the contrasting rearing systems were associated with further variation in host-associated microbial communities [1,2,25]. Under this framework, the intestine showed stronger niche selectivity and regional divergence, whereas the gill retained a closer, though still selective, relationship with the surrounding water microbial community [8,32,33]. These findings deepen current understanding of large yellow croaker microbiome ecology and provide a theoretical basis for future work on microbiome manipulation, probiotic development, and environmental optimization in different aquaculture systems [5,26].

Several limitations should also be acknowledged. First, each host-associated group contained three composite biological replicates, and each composite sample was generated by pooling tissues from three fish within the same culture unit. This design reduced the influence of extreme individual variation and allowed comparison at the culture-unit level, but it also masked inter-individual variability and limited the statistical power for detecting subtle microbial differences. Therefore, the present findings should be interpreted as preliminary ecological patterns associated with host mucosal niche and rearing environment. Future studies should include larger sample sizes, individual-level microbiome profiling, and controlled rearing experiments to further validate the observed patterns.

## 5. Conclusions

In conclusion, this study showed that the gut and gill microbiota of large yellow croaker differed markedly between host mucosal niches and the marine and saline–alkaline rearing systems. Both gut and gill samples harbored distinct microbial assemblages that differed clearly from the surrounding water microbial community, indicating host-associated microbial differentiation. At the same time, marked differences between the two rearing systems suggested that culture conditions were associated with additional variation in host-associated microbiota. This divergence became more pronounced at finer taxonomic resolution, highlighting the sensitivity of fish-associated microbial communities to rearing-system-associated variation.

The gut microbiota exhibited stronger marine and saline–alkaline rearing system differentiation than the gill microbiota, suggesting greater niche selectivity in the gut habitat, whereas the gill microbiota retained a closer, though still selective, association with the surrounding water microbial community. In addition, significant correlations between dominant taxa and environmental variables, especially salinity and nutrient-related factors, suggested that physicochemical heterogeneity was linked to microbial community variation. Several taxa with relatively high network topological importance were also identified, providing preliminary candidates for future functional validation rather than direct evidence of ecological interactions or probiotic potential.

Overall, our findings provide preliminary microbial ecological evidence for understanding microbiota variation in large yellow croaker under contrasting culture conditions. These results are relevant to the emerging “marine fish cultured on land” model because they suggest that alternative rearing systems may be associated with shifts in host-associated microbial communities. However, further controlled experiments and functional validation are required before these findings can be translated into practical microbiome-based management strategies for land-based marine aquaculture.

## Data Availability

The original contributions presented in the study are included in the article/Supplementary Materials. Further inquiries can be directed to the corresponding author.

## Supplementary Materials

The following supporting information can be downloaded at https://www.mdpi.com/article/10.3390/vetsci13070710/s1: Figure S1: Microbial community composition at class level; Figure S2: Microbial community composition at order level; Figure S3: Microbial community composition at family level; Figure S4: Venn diagram analysis of microbial differences among 4 water samples; Figure S5: Venn diagram analysis of inter-microbial differences in the gut; Figure S6: Venn diagram analysis of microbial differences in fish gills; Table S1: Sampling information, physical and chemical parameters, nutrient table, and Envfit analysis table of environmental variables associated with microbial community structure based on db-RDA; Table S2: Spearman correlation heatmap table; Table S3: Genus-level structural keystoneness results based on correlation-derived topological analysis; Table S4: Zi–Pi classification of ASV nodes based on significant microbial associations.
