# Supplementary material for: Host Mucosal Niche and Rearing Environment Are Associated with Distinct Gut and Gill Microbiota of L. crocea (Larimichthys crocea)
Source: Vet Sci. 2026 Jul 19;13(7):710. doi: 10.3390/vetsci13070710 (PMC13419284; doi:10.3390/vetsci13070710)
Supplement: Supplementary file 1 [file vetsci-13-00710-s001.zip › Supplementary Figure_Host niche and rearing environment are associated with dis-tinct gut and gill microbiota of L. crocea (Larimichthys crocea).pdf]

veterinary sciences

MDPI

Supplementary Material

Supplementary Figures

FIGURE S1 Microbial community composition at class level.

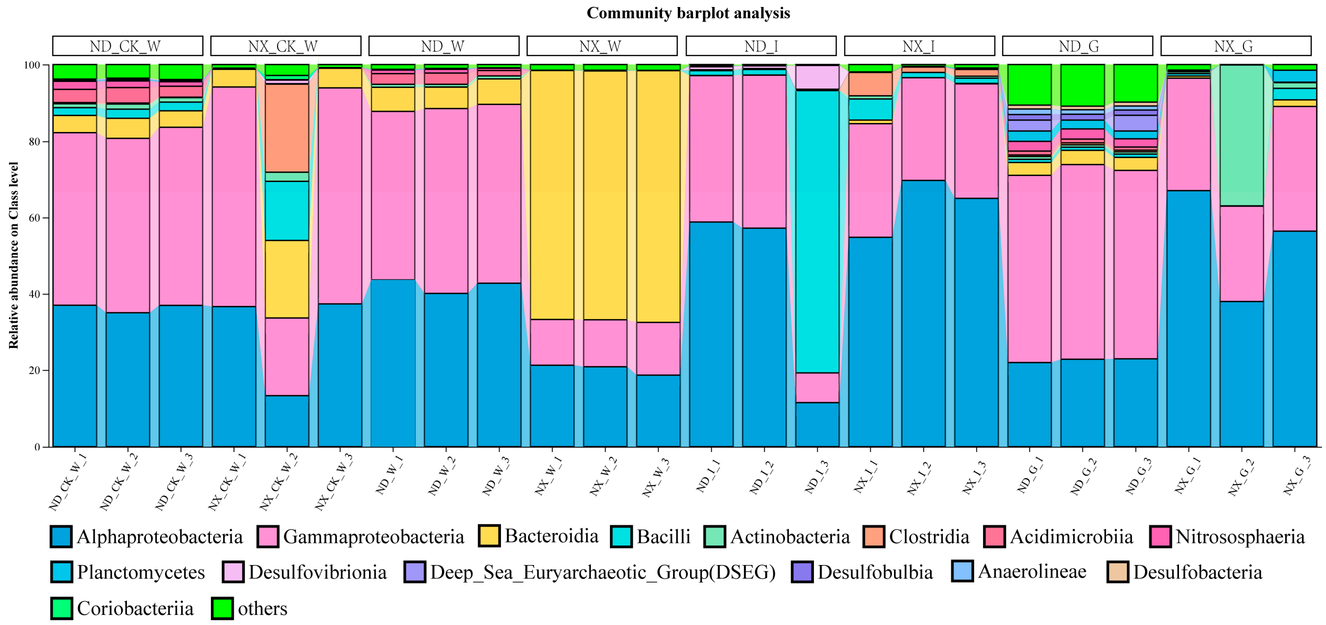

FIGURE S2 Microbial community composition at order level.

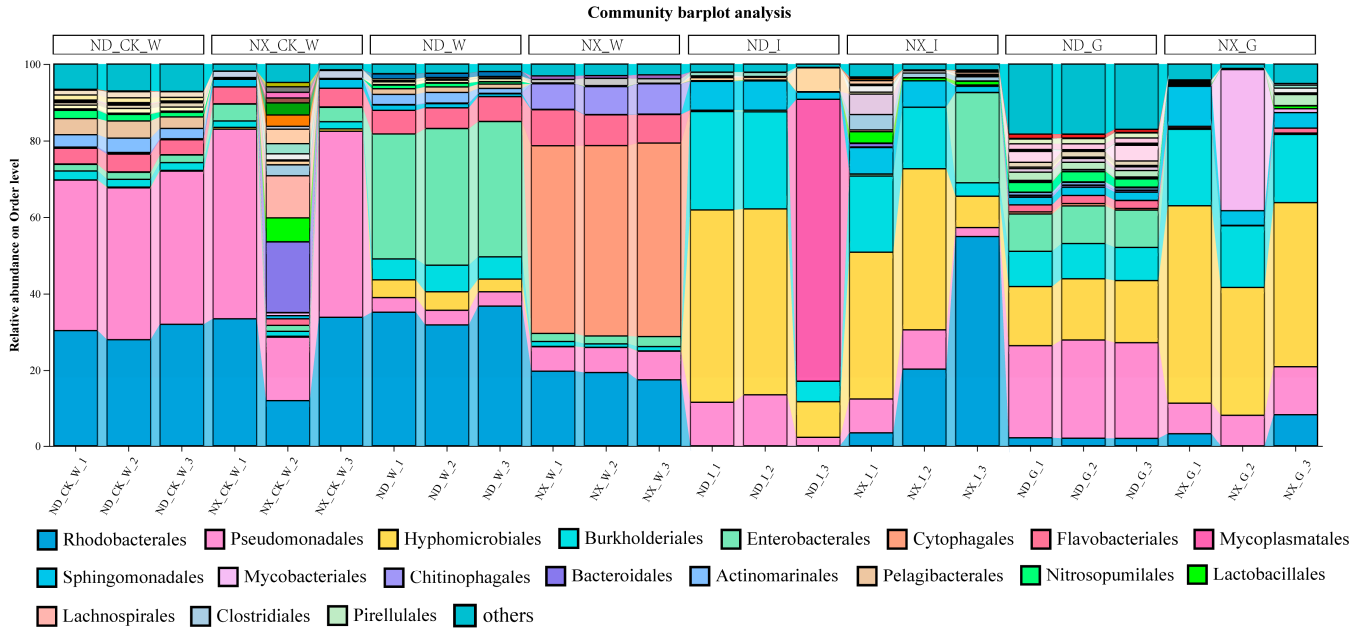

FIGURE S3 Microbial community composition at family level.

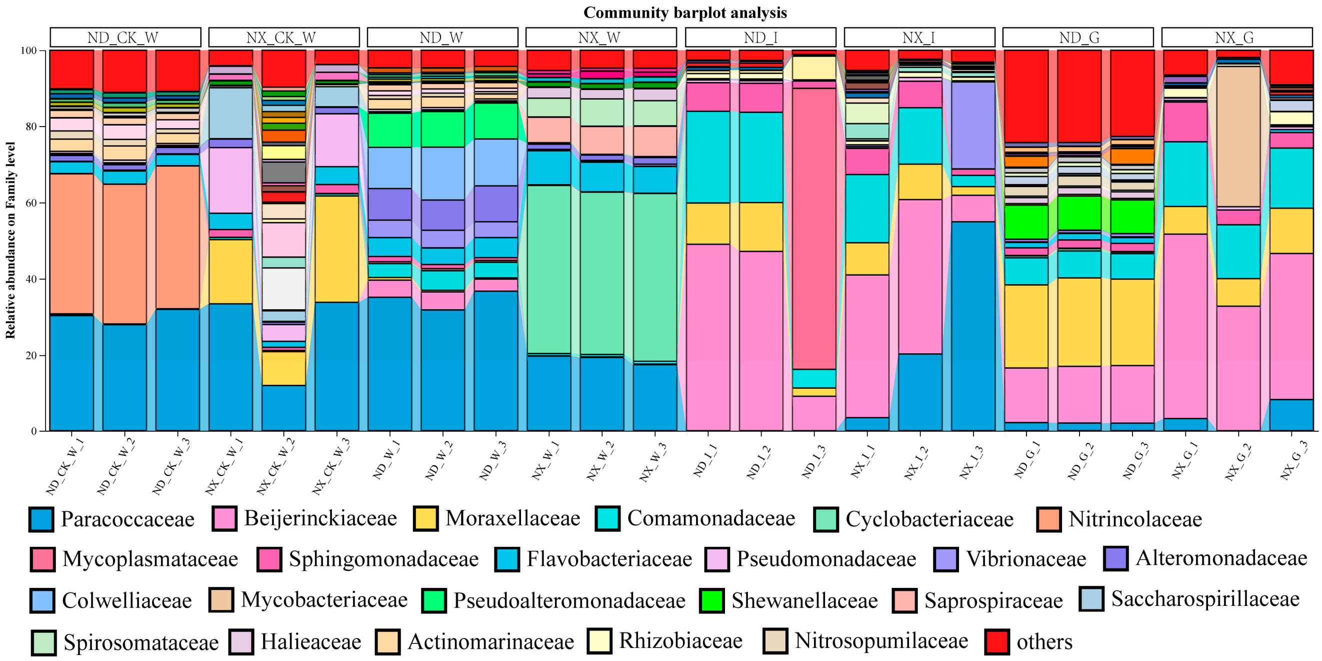

FIGURE S4 Venn diagram analysis of microbial differences among 4 water samples. (A) phylum level, (B) family level, (C) genus level, (D) ASV level.

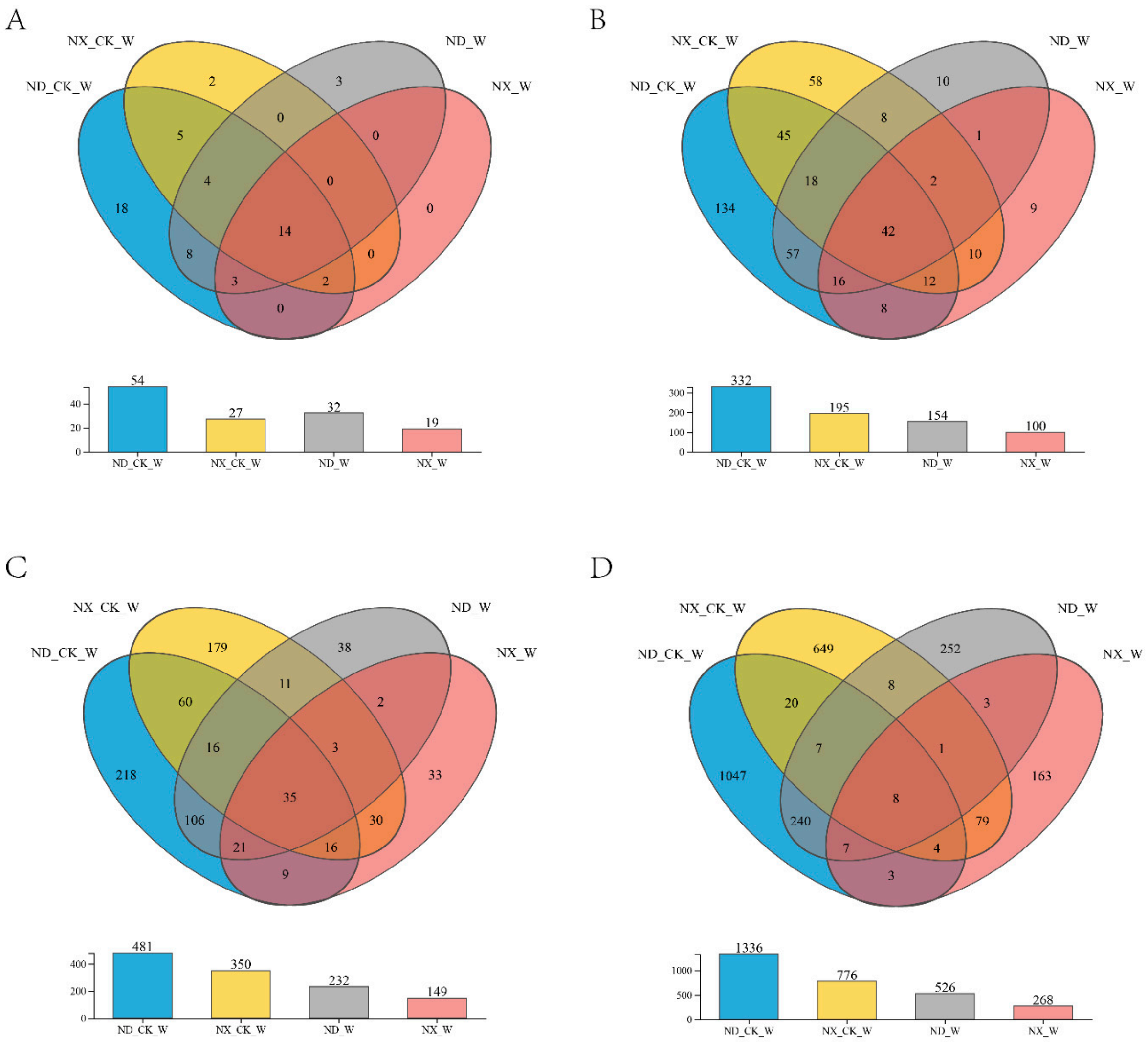

FIGURE S5 Venn diagram analysis of inter-microbial differences in the gut.
(A) phylum level, (B) family level, (C) genus level, (D) ASV level.

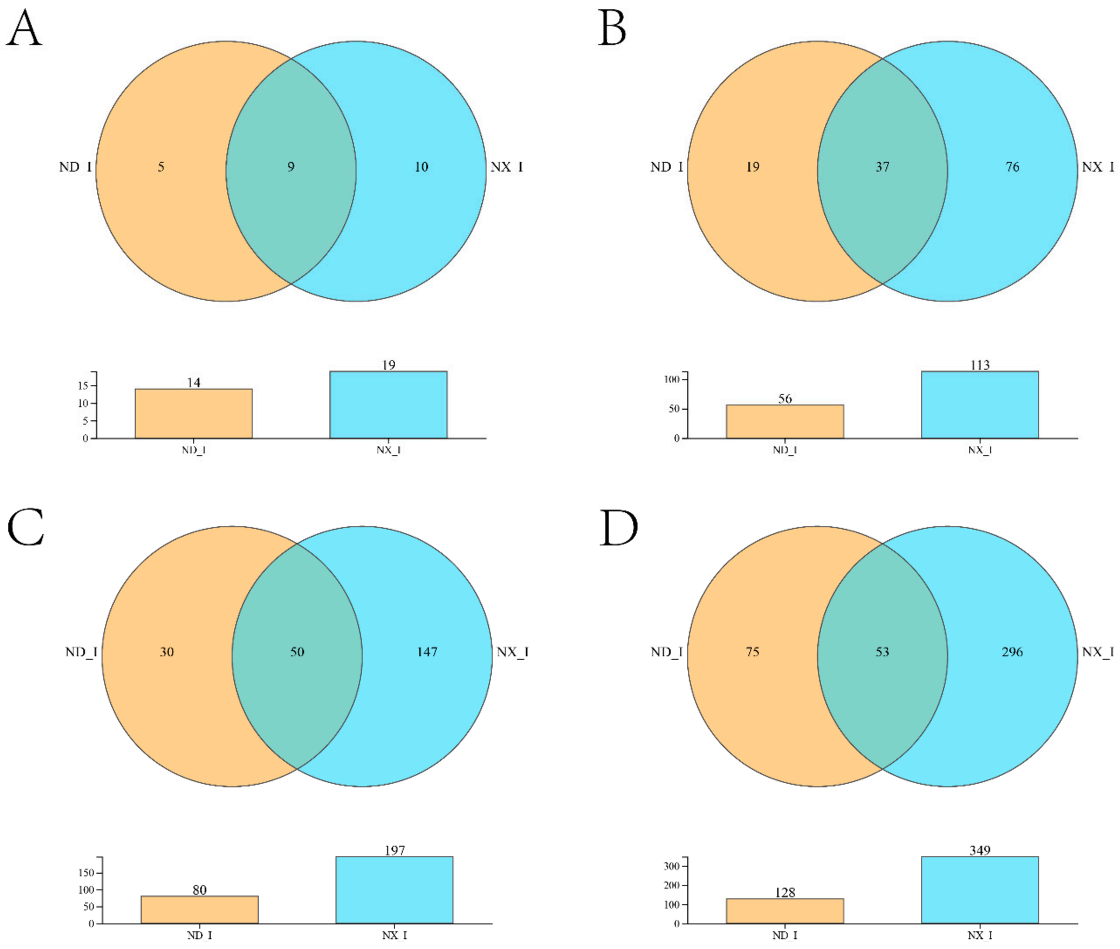

FIGURE S6 Venn diagram analysis of microbial differences in fish gills.
(A) phylum level, (B) family level, (C) genus level, (D) ASV level.

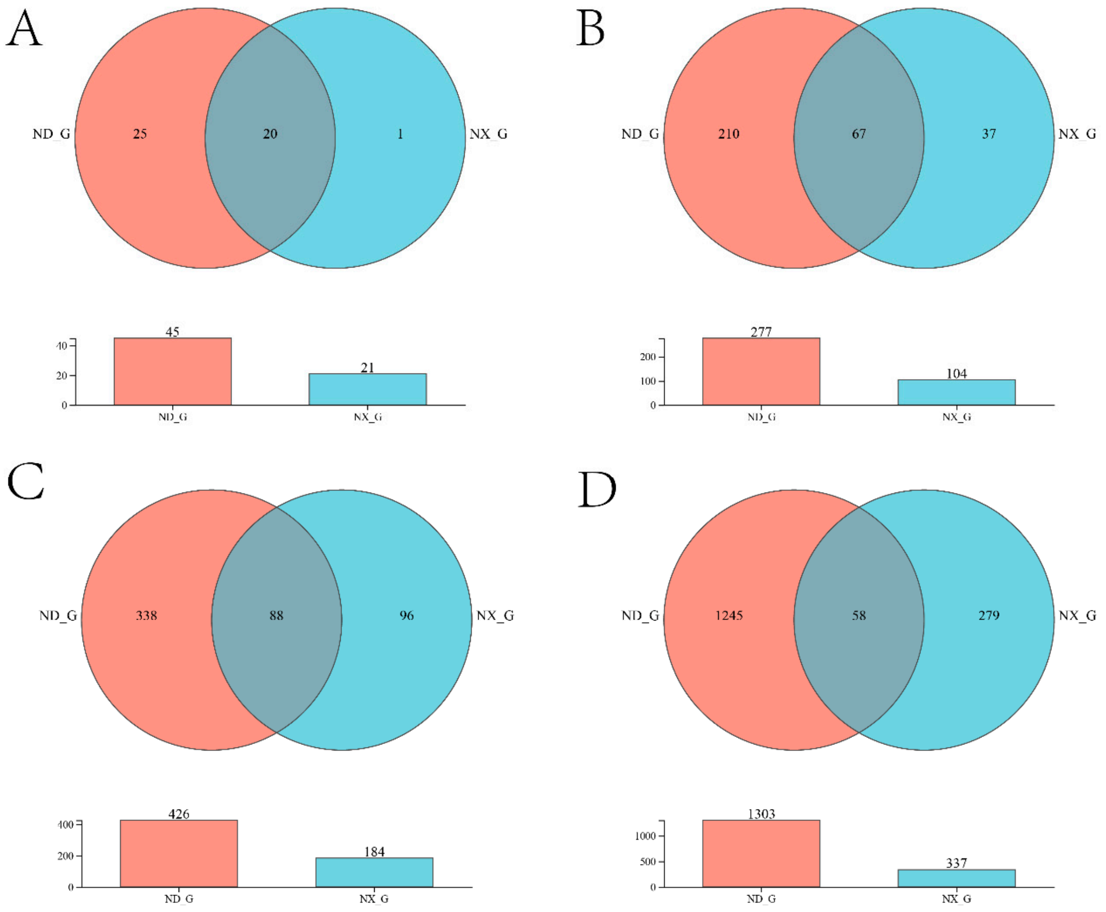
A
ND_G
25
20
1
NX_G
45
21
20
0
ND_G
NX_G
B
ND_G
210
67
37
NX_G
277
104
200
100
0
ND_G
NX_G
C
ND_G
338
88
96
NX_G
426
184
200
0
ND_G
NX_G
D
ND_G
1245
58
279
NX_G
1303
337
1000
500
0
ND_G
NX_G
